# Relationship between Cervical Spine and Skeletal Class II in Subjects with and without Temporomandibular Disorders

**DOI:** 10.1155/2018/4286796

**Published:** 2018-10-16

**Authors:** Paola Di Giacomo, Valeria Ferrara, Ettore Accivile, Giacomo Ferrato, Antonella Polimeni, Carlo Di Paolo

**Affiliations:** Department of Oral and Maxillofacial Sciences, Sapienza University of Rome, Rome, Italy

## Abstract

**Aim:**

To assess changes in the craniocervical structure and in hyoid bone position in skeletal Class II subjects with and without temporomandibular disorders (TMD).

**Materials and Methods:**

The cephalometric analysis of 59 subjects with skeletal Class II was evaluated and compared. The measurements considered were ANB as a parameter of Class II and C0-C1 distance, C1-C2 distance, craniocervical angle, and hyoid bone position for the cervical spine analysis. Patients were divided into patients with TMD (group A) and patients without TMD (group B). TMD were evaluated with Diagnostic Criteria for TMD (DC/TMD). Descriptive statistics and Pearson's and Spearman's correlation analysis, with *p* value <0,005, were performed.

**Results:**

C0-C1 and C1-C2 distance values and hyoid bone position resulted within the normal range in the majority of patients examined. Craniocervical angle was altered in 33 patients. The reduction of this angle with the increase of the ANB value resulted to be statistically significant in group A, according to Pearson's correlation index. No other data were statistically significant.

**Conclusions:**

The significant relationship between skeletal Class II and cervical spine cannot be highlighted. The alteration of craniocervical angle seems to be mildly present, with backward counterclockwise rotation of the head upon the neck in the sample (groups A and B). The presence of TMD as a key factor of changes in neck posture could explain the different result between the two groups about the relationship between ANB and craniocervical angle. This result should be further analyzed in order to better understand if cervical spine changes could be related to mandibular postural ones in the craniocervical space or to temporomandibular joint retropositioning, more recognizable in Class II with TMD, which could determine functional changes in other structures of this unit; neck posture could be the result of a compensatory/antalgic mechanism in response to TMD.

## 1. Introduction

The stomatognathic system is composed of several structures which, acting in synergy, provide the complex functions of swallowing, sucking, chewing, speech, and breathing. Functionality of these components has a cybernetic and integrated control, and it depends on the specific individual adaptability and response to perturbing factors. This system correlates with the cervical spine and hyoid bone, forming a functional craniocervical mandibular unit. For this reason, the principles of head and neck biomechanics are of great interest both in the dental field (particularly in the orthodontic and gnathologic field) and in the physical rehabilitation one.

Because of the impossibility to record functions in the same moment in which they are performed, anatomical relationships among the examined structures are analyzed in order to evaluate functional and dysfunctional aspects of the craniocervical mandibular system.

Many studies found support for anatomical [1, 2] and functional [3, 4] relationship between the cranial region, the temporomandibular joint, the cervical spine, and the hyoid bone. Although there is presence of suggestive results in these studies, the mechanism of this relationship is still unclear.

In the literature, cervical posture is related to different factors of the body such as craniofacial morphology [5, 6] and functional factors such as nasorespiratory function [7] and temporomandibular dysfunction [2]. Previous studies were focused on the relationship between the cervical spine and skeletal class of the subjects [2, 5, 6], but results were contrasting. Skeletal Class II malocclusions were chosen for this study, needing to analyze a specific skeletal category to compare with data obtained in the literature [6, 8, 9] and needing to have a homogeneous sample under the point of view of skeletal class, within which a large subgroup of dysfunctional patients could be collected. In fact, skeletal Class II subjects are more easily linked to temporomandibular disorders [10, 11].

The choice to deal also with TMD is because the relationship between temporomandibular joint “status” and head-neck posture should be investigated, within the craniocervical mandibular unit. Temporomandibular disorders are often associated with headache and neck pain. The relation between TMD and the head and neck posture changes is still controversial and unclear [12]. Some studies [13, 14] found correlation between the presence of symptoms of craniocervical dysfunction and temporomandibular disorders. On the contrary, others [11, 15] found no relationship between TMD and head-neck posture.

In this study, means that are scientifically appropriate, easy to use, and produce measurable results were used. For this reason, data were gathered from cephalometric analysis, carried out on lateral X-ray of the skull (lateral skull radiographs) which, together with orthopantomography, represents basic X-ray imaging in orthodontic and gnathologic fields. In fact, this allows to evaluate possible changes in the physiological curve of the cervical spine, head position, and hyoid bone position, in addition to standard orthodontic skeletal assessment.

## 2. Materials and Methods

### 2.1. Study Design

To address the research purpose, patients were recruited among those who came under our observation at the Gnathology Section of the Department of Oral and Maxillo-facial Sciences, Sapienza University of Rome, and at the Clinical Operative Unit of Orthodontics of the George Eastman Hospital, Sapienza University of Rome, between October 2017 and March 2018, according to the inclusion and exclusion criteria.

The study was approved by the Institutional Human Ethics Committee, Sapienza University of Rome.

#### 2.1.1. Inclusion Criteria

- Patients with presence of skeletal Class II
- Patients at least 18 years old
- Patients who provided signed informed consent, according to the World Medical Association's Declaration of Helsinki

#### 2.1.2. Exclusion Criteria

- Loss of posterior teeth
- History of trauma
- Previous orthodontic and/or gnathologic and/or physical therapies
- Presence of further structural malformations in the areas of interest
- Presence of uncontrolled systemic disease

On the basis of these criteria, the sample comprised 59 individuals, 38 females and 21 males, with an average age of 33.65 years.

The sample was divided into two subgroups: group A—patients with dysfunctions, and group B—patients without dysfunctions. The integrated DC/TMD method was used for the screening of temporomandibular disorders [16] and to assess which patients belonged to group A and which to group B. Group A comprised 26 patients, 24 females and 2 males, with an average age of 44.69 years. Only patients with the following disorders were considered: disc displacement with reduction; myalgia and myofascial pain; subluxation; headache associated with TMD; arthralgia; and osteoarthrosis. Disc displacements without reduction were not taken into account because the complexity of pathology has many confounding factors.

Group B comprised 33 individuals, 14 females and 19 males, with an average age of 24.33 years.

### 2.2. Cephalometric Assessment

All cephalograms were made upon a standardized lateral radiograph (18 Å∼24 cm film; Kodak, Germany) on the basis of natural head position (patients' midplane-X-ray source distance 146 cm; patients' midplane-film distance 13.5 cm; enlargement factor 1%; and exposure 4–21 mAs, 60–80 kV) by a single technician in the Radiology Unit of George Eastman Hospital, Policlinico Umberto I, Rome.

Specific linear and angular measures from the cephalometric analysis performed on the lateral skull radiographs were assessed. The cephalometric analysis used was chosen among those proposed in the literature and scientifically validated for the study of the relationships between skull bones, in particular jaw and mandible, and between these and the cervical spine and the hyoid bone. McNamara's modified cephalometric analysis was used, and linear and angular measures were performed. Among the various affected measures, the cephalometric parameter considered for the evaluation of presence of skeletal Class II was ANB angle, according to Steiner (normal value 2° ± 2°) [17].

The standard cephalometric analysis was associated with the method proposed by Rocabado [18] for the study of the other cervical parameters. The parameters considered in the study were as follows:

- The craniocervical angle which is the inner angle formed by the intersection of McGregor's plane (line passing through the occiput and nasal spine) and odontoid plane (line passing through the inferior-anterior point of the odontoid process and its superior apex) with a normal value of 101 ± 5.
- The C0-C1 vertebrae distance with a normal range of 6.5 ± 2.5 mm.
- The C1-C2 vertebrae distance with a normal range of 6.5 ± 2.5 mm.
- Hyoid bone position defined by the relationship between the H point (superoanterior point of the hyoid bone) and Hʹ line (conjunction line between retrognathion and C3 vertebra). The distance H-Hʹ should be 5 ± 2 mm.

### 2.3. Statistics

Data taken from the cephalometric analysis were interpreted using basic descriptive statistical analysis and Pearson's correlation index for random variables and the nonparametric Spearman's ordinary measures to evaluate the correlation between parameters.

#### 2.3.1. Correlation Scores

- 0 < *ρ*_*XY*_ < 0.3–weak correlation
- 0.3 < *ρ*_*XY*_ < 0.7–moderate correlation
- *ρ*_*XY*_ > 0.7–strong correlation

The level of significance (*p* value) is 0.005, verified according to Student's *t* tables (for Pearson's correlation results and Spearman's correlation analysis with *N* > 30) and according to Spearman's rho value for Spearman's correlation analysis with *N* < 30.

## 3. Results

Data obtained were primarily organized in tables and analyzed with descriptive statistics. Only significant results were presented, considering the great amount of data.

The absolute frequency distribution of each ANB value (5 to 9°/10°) was reported. For each ANB value, the absolute and percent frequency distribution of the values of the measured cervical variables, divided on the basis of the severity as abnormally low, normal, and abnormally high, was considered.

Furthermore, these results were divided into two subgroups, on the basis of the presence or not of temporomandibular disorders. Percent frequencies of DC/TMD diseases are reported in Table 1.

For what concerning skeletal Class II and cervical alterations, independently from the presence of temporomandibular disorders, the following results were found: 65% of all subjects examined (38 subjects) showed normal values of C0-C1 space on the vertical plane. The C1-C2 space was normal in 73% of the patients (43 subjects), as shown in Tables 2 and 3. The hyoid bone was found to be in a normal limit position in 51% (30 subjects) of the subjects examined and in an outside positive position in 49% of the cases (29 subjects) (Table 4).

Craniocervical angle measurement was out of standard in 56% of the patients (33 subjects); 13 of them (40%) were found to be positive to TMD. It was reduced in 51% of the patients (30 subjects), normal in 44% (26 subjects), and increased in 5% of them (3 subjects) (Table 5).

In our work, the descriptive analysis found that 35.60% of the patients presented an ANB angle of 5° (21), and these subjects showed the highest number of abnormal values of all cervical measurements considered.

For what concerning skeletal Class II, temporomandibular disorders, and cervical anomalies, the C0-C1 space was altered in 30% of not-dysfunctional patients and in 42% of dysfunctional patients and the C1-C2 space was altered in 27% of both not-dysfunctional and dysfunctional patients. Craniocervical angle in not-dysfunctional patients showed some anomalies in 20 of 33 subjects (61%), while in the dysfunctional group, it was altered in 13 of 26 subjects (50%). Hyoid bone position was altered in 54% of the dysfunctional patients (14 subjects); this result was similar in 45% of nondysfunctional subjects (15 of 18). There are not significant percent differences between the two groups.

There are no statistically significant data (Tables 6–8) except for the moderate inverse correlation between the increase of ANB and the reduction of craniocervical angle in the dysfunctional group, in Pearson's correlation test (Table 9).

## 4. Discussion

From the analysis of the entire sample, there were not particular significant results in percentage terms for what concerning the relationship between skeletal Class II in adult patients and neck structure and posture, within the limits of the sample examined. The statistical correlations did not show significance between the variables, either in positive or in negative sense. Correlation indexes were always very close to zero, and these proved that distributions were independent of the previous associations. This is not in line with the results obtained in some publications of the scientific literature, which support a correlation between skeletal class II and morphologic and postural cervical spine alterations [6, 9, 19]. Festa [6] found the relationship between cervical lordosis and cranial base and mandibular length. Hosseinzadeh Nik and Aciyabar [9] found significant correlation between cervical column posture angles and the parameters ANB and Wits in Class II patients. Sonnessen [18] found associations between fusions of the cervical column and mandibular retrognathia, large cranial base angle, and large horizontal overjet, and Miyuki et al. [20] found that Class II subjects have significantly lower atlas dorsal arch heights. On the contrary, Bebnowski et al. [21] found that cervical anomalies (CVAs) did not correlate to any cephalometric values nor they could be confirmed by CBCT, the gold standard for assessing CVA.

These studies represented the starting point of this research which should be verified in our sample. The data obtained do not confirm what has been said in these previous research studies. The only alteration which can be reported is about craniocervical angle, but it is present only in 56% of the patients, so it cannot be considered to be representative.

Significance is the inverse correlation, according to Pearson's analysis, between the increase of ANB and the reduction of craniocervical angle. These data are not recognizable in the entire sample, but only in the dysfunctional subgroup. The different result between the two groups could be explained by the fact that the factor related to postural neck changes is not so much represented by the sagittal skeletal pattern of the jaws but the presence of temporomandibular disorders. The relationship between TMD and cervical spine is debated, but cervical painful symptomatology is often referred by dysfunctional patients. The mechanism underlying these data is probably linked to close spatial relationships among these structures [22, 23] and due to the fact that they are parts of the same functional unit [23].

There are cephalometric parameters that should be investigated to better understand the results obtained in this study and to verify that there are not other differences between the two groups, except for temporomandibular disorders. A comparison between SNA and SNB values in order to evaluate the prevalent component of Class II in each group should be done; a comparison between SNB and mandibular length in both groups is needed in order to analyze the presence of a retropositioned mandible or of reduction of the lengthy one. Lippold et al. [24] argue that the mandible seems to have a greater effect on body posture than other craniofacial parameters. Some studies [2, 6] report the relationship between mandibular length and cervical posture. However, from what emerged from Pearson's correlation analysis, the mandibular component of Class II more often associated with TMD should be analyzed. And this component is the retropositioning of the mandible, as said in the literature [25]. Retropositioned mandible, which could be the cause or effect of TMD itself, is associated with Class II and linked to the increase of ANB value and of severity of TMD, as said previously [25]. The increased severity of temporomandibular disorders also could lead to a compensatory or antalgic posture of the neck, as confirmed by the literature [2].

The anatomical change in a specific district could not justify the anatomical change in other districts. Instead functional/dysfunctional changes, determined by “anatomical links” such as muscles, ligaments, and joints, could influence other structures. The analysis of Class II components could be important in order to verify that it is not the mandibular length or the maxillary protrusion in skeletal Class II but the spacial postural changes in the mandible, such as retropositioning or divergence, which could lead to other changes. These do not necessarily have the pathologic mean as seen in this study; in fact, also in the dysfunctional group, only 40% had an alteration of craniocervical angle.

## 5. Conclusions

The findings of this study, within the limits of the sample, may be useful to understand that significant data are not emerged in relation to Class II and cervical spine alterations in both groups. Structures should be better investigated under a functional point of view, although there is an intrinsic limit for the record of the functional aspects.

Cervical spine assessment could be influenced by functional changes in the mandible, rather than the anatomical one, but further studies in larger samples and according to previous indications are requested. Anyway, these changes do not seem to have a great pathologic mean from what have been emerging from the study.

## Figures and Tables

**Table 1:** Absolute frequency and percentage (*n* (%) values) of DC/TMD diseases.

DC/TMD disease	Prevalence (%) (*N* = 26)
Muscle/myofascial pain	50 (13)
Arthralgia	82 (21)
Disc dislocation with reduction	8 (2)
Subluxation	13 (3)
Headache associated with TMD	31 (8)
Osteoarthrosis	8 (2)

**Table 2:** Absolute and percent frequencies of the C0-C1 distance value for each ANB score. ANB scores are 5 to 9°/10°. C0-C1 values are divided into abnormally low, normal, and abnormally high. Class II is divided on the basis of the presence or not of TMD.

TMD	ANB	C0-C1 space			
	Value	Abnormally low (<4.5 mm)	Normal	Abnormally high (>4.5 mm)	Total.
Absent	5	2	14	1	17
	6	1	2	5	8
	7	0	3	1	4
	9	0	4	0	4
	Total	3	23	7	33
		0.09	0.70	0.21	1.0

Present	5	6	8	0	14
	6	3	4	2	9
	7	0	1	0	1
	9	0	1	0	1
	10	0	1	0	1
	Total	9	15	2	26
		0.35	0.58	0.08	1.0

**Table 3:** Absolute and percent frequencies of the C1-C2 distance value for each ANB score. ANB scores are 5 to 9°/10°. C1-C2 values are divided into abnormally low, normal, and abnormally high. Class II is divided on the basis of the presence or not of TMD.

TMD	ANB	C1-C2 space			
	Value	Abnormally low (<4.5 mm)	Normal	Abnormally high (>4.5 mm)	Total
Absent	5	2	11	4	17
	6	2	5	1	8
	7	0	4	0	4
	9	0	4	0	4
	Total	4	24	5	33
		0.12	0.73	0.15	1.0

Present	5	3	11	0	14
	6	3	5	1	9
	7	0	1	0	1
	9	0	1	0	1
	10	0	1	0	1
	Total	6	19	1	26
		0.23	0.73	0.04	1.0

**Table 4:** Absolute and percent frequencies of hyoid bone position for each ANB score. ANB scores are 5 to 9°/10°. Values are divided into abnormally low and normal. Class II is divided on the basis of the presence or not of TMD.

TMD		ANB	Hyoid bone position		
		Value	Abnormally low (<3 mm)	Normal	Total
Absent		5	8	9	17
		6	5	3	8
		7	1	3	4
		9	1	3	4
		Total	15	18	33
			0.45	0.55	1.0

Present		5	7	7	14
		6	6	3	9
		7	1	0	1
		9	0	1	1
		10	0	1	1
		Total	14	12	26
			0.54	0.46	1.0

**Table 5:** Absolute and percent frequencies of the craniocervical angle value for each ANB score. ANB scores are 5 to 9°/10°. Values are divided into abnormally low, normal, and abnormally high. Class II is divided on the basis of the presence or not of TMD.

TMD	ANB	Craniocervical angle			
	Value	Abnormally low (<96°)	Normal	Abnormally high (>106°)	Total
Absent	5	8	7	2	17
	6	4	4	0	8
	7	3	1	0	4
	9	3	1	0	4
	Total	18	13	2	33
		0.55	0.39	0.06	1.0

Present	5	6	7	1	14
	6	4	5	0	9
	7	0	1	0	1
	9	1	0	0	1
	10	1	0	0	1
	Total	12	13	1	26
		0.46	0.50	0.04	1.0
		1.0	1.0	1.0	1,0

**Table 6:** Pearson's and Spearman's correlation analysis between C0-C1 distance and ANB scores.

TMD	Pearson's correlation index	Student's *t* test (*p* < 0.005)	Spearman's correlation index	Student's *t* test (*p* < 0.005)
Absent (*N* = 33)	0.147 (L)	0.830 (NS)	0.158 (L)	0.890 (NS)

Present (*N* = 26)	0.113 (L)	0.5 (NS)	0.152 (L)	NS according to critical values table for the nonparametric test

L: low correlation; NS: nonsignificant. Student's *t* test is used for the evaluation of significance, with a *p* value <0.005.

**Table 7:** Pearson's and Spearman's correlation analysis between C1-C2 distance and ANB scores.

TMD	Pearson's correlation index	Student's *t* test (*p* < 0.005)	Spearman's correlation index	Student's *t* test (*p* < 0.005)
Absent	0.043 (L)	0.241 (NS)	0.100 (L)	0.560 (NS)

Present	−0.039 (L)	−0.190 (NS)	0.134 (L)	NS according to critical values table for the nonparametric test

L: low correlation; NS: nonsignificant. Student's *t* test is used for the evaluation of significance, with a *p* value <0.005.

**Table 8:** Pearson's and Spearman's correlation analysis between hyoid bone position and ANB scores.

TMD	Pearson's correlation index	Student's *t* test (*p* < 0.005)	Spearman's correlation index	Student's *t* test (*p* < 0.005)
Absent	0.024 (L)	0.135 (NS)	0.072 (L)	0.403 (NS)

Present	−0.109 (L)	−0.535 (NS)	−0.145 (L)	NS according to critical values table for the nonparametric test

L: low correlation; NS: nonsignificant. Student's *t* test is used for the evaluation of significance, with a *p* value <0.005.

**Table 9:** Pearson's and Spearman's correlation analysis between craniocervical angle and ANB scores.

TMD	Pearson's correlation index	Student's *t* test (*p* < 0.005)	Spearman's correlation index	Student's *t* test (*p* < 0.005)
Absent	−0.085 (L)	−0.475 (NS)	−0.169 (L)	−0.954 (NS)

Present	−0.547 (M)	−3.198 (S)	−0.212 (L)	NS according to critical values table for the nonparametric test

L: low correlation; M: moderate correlation; NS: nonsignificant. Student's *t* test is used for the evaluation of significance, with a *p* value <0.005.

## Data Availability

The cephalometric and statistical data used to support the findings of this study are included within the article.
